# Computational Simulation and Experimental Validation of Acoustic Reflectometry in Otitis Media

**DOI:** 10.3390/s25247420

**Published:** 2025-12-05

**Authors:** Karl Nyberg, Manfred Lindmark, Mimmi Werner, Petter Holmlund, Thorbjörn Lundberg, Fredrik Öhberg

**Affiliations:** 1 Departament of Diagnostics and Intervention, Biomedical Engineering and Radiation Physics, Umeå University, 90187 Umeå, Sweden; kany0097@student.umu.se (K.N.);; 2 Departament of Clinical Sciences, Otorhinolaryngology, Umeå University, 90187 Umeå, Sweden; 3 Departament of Applied Physics and Electronics, Umeå University, 90187 Umeå, Sweden; 4 Departament of Public Health and Clinical Medicine, Family Medicine, Umeå University, 90187 Umeå, Sweden

**Keywords:** otitis media, acoustic reflectometry, middle ear fluid, computational simulation, primary care diagnostics, experimental modelling

## Abstract

Otitis Media (OM) is a prevalent condition in children that can lead to hearing impairment and significant healthcare costs. Inaccuracy in primary care and equipment cost in developing countries are concerning issues in OM diagnostics. Acoustic Reflectometry (AR) offers a low-cost, non-invasive diagnostic alternative, though it has fallen short on accuracy in previous studies. The primary aim of this study was to establish a computational simulation and an experimental model able to reproduce AR performed on human individuals to enable further research and accuracy improvement. The secondary aim was to perform a sensitivity analysis on AR instrument user error. Simulations and experiments were validated against measurements from human individuals with OM and normal ears, respectively. The results reveal that the simulation sufficiently reproduces human AR measurements and distinguishes an ear with OM from a healthy ear. The experiment delivered satisfying measurements on OM but underperformed in a normal ear scenario. The simulations and experiments overpredicted sound reflection in OM. The sensitivity study showed promising robustness of AR, concluding that computational simulation is a viable tool and complement to an experimental approach in research of AR. Future efforts should focus on paediatric models and partially filled middle ear simulations to promote clinical relevance.

## 1. Introduction

Ear infections are common in children all over the world, and by the age of three, 80% will have experienced at least one episode of Otitis Media [1]. Otitis Media (OM) includes three different diagnoses: Acute Otitis Media (AOM), Otitis Media with Effusion (OME), and Chronic Suppurative Otitis Media (CSOM). In AOM, the middle ear is invaded by bacteria, resulting in purulent secretion, inflammation, swelling, and blocking of the eustachian tube that will result in increased pressure in the middle ear and bulging of the tympanic membrane (TM). Typical symptoms of AOM are fever, pain, and temporary hearing loss. Correct diagnosis, follow-up, and adequate treatment at the right time are crucial to avoiding serious complications such as mastoiditis and meningitis, and to preventing consequences such as deafness and death. Not only are these complications tragic for the individual, but they also pose a significant economic burden for society [2,3]. Choice of treatment of AOM is a complex task where condition severity, symptoms, timing, patient history, and age decide whether antibiotics are appropriate. Many European countries have guidelines regarding treatment of AOM to prevent complications and maintain restrictive antibiotic treatment to avoid contribution to the growing problem of antibiotic resistance. In Sweden, for instance, children between 1 and 12 years old with uncomplicated AOM are not eligible for antibiotic treatment before 2–3 days of watchful waiting [4]. AOM is often followed by OME, in which the infection has resolved but inflammation persists, keeping the eustachian tube closed and allowing fluid to accumulate due to transudation. Opposite to AOM, the middle ear pressure turns negative in OME, recognised by a retracted TM. OME should not be treated with antibiotics, but instead with instructions to facilitate ventilation of the middle ear and evacuation of the serous fluid, and eventually ventilation tubes. If recurrent or inadequately treated, AOM and OME can progress to CSOM where the TM is perforated, allowing pathogens to enter from the ear canal and cause further damage to auditory structures. Treatment of CSOM is selected based on severity and preceding condition, ranging from watchful waiting to antibiotics or otosurgery. Correct diagnosis is crucial for optimal treatment in all forms of OM to avoid serious complications and death and also to limit the development of antibiotic resistance in society. Diagnostic inaccuracies in primary care are an alarming problem, with disagreement rates of up to 20% between general practitioners and otorhinolaryngologists [3]. Limited access to trained personnel and diagnostic equipment is a major barrier in developing countries [1], while training and diagnostic guidelines are areas of improvement for developed countries [3]. Around 10 million children suffer from hearing impairment caused by ear infections, and up to 60% of these cases are believed to have been preventable [1].

Otoscopy is a fundamental instrument allowing General Practitioners (GPs) to diagnose ear infections [5], based on visual inspection of the ear canal and tympanic membrane (TM). Although otoscopy plays a central part in diagnosing OM, it needs complementary diagnostics to reach sufficient accuracy [5,6]. Pneumatic otoscopes are purposefully built to reveal fluid and pressure change in the middle ear, which both manifest with decreased TM mobility. In pneumatic otoscopy, a rubber bulb is used to increase pressure momentarily in the air of the ear canal; a healthy TM will respond in a yielding fashion, while little or no movement is observed in cases of AOM or OME. Proper training is required to master this instrument, and even though it increases diagnostic accuracy in AOM and OME significantly compared to regular otoscopy, it is scarcely used among GPs [5]. Tympanometry is an acoustic method that uses sound reflection to assess TM mobility and presents high accuracy in detecting middle ear fluid (MEF) and changes in middle ear pressure causing TM tension [7]. However, the device probe requires an airtight seal and is not always tolerated by paediatric patients [8], causing GPs to refrain from using it [5]. The equipment is also quite expensive, thereby making it unavailable in developing countries. Acoustic Reflectometry (AR) instruments, such as EarCheck (Innovia Medical LLC, Omaha, NE), is a low-cost acoustic method and does not require an airtight fit, though it comes up short in terms of accuracy compared to tympanometry [9,10,11]. More specifically, it cannot distinguish the purulent MEF of AOM, secretory MEF of OME, or changes in middle ear pressure. However, looking at the user friendliness and economic traits of AR, there are scenarios where AR can be a good contribution to OM diagnostics [12,13]. Even parental use can be considered, which could significantly alleviate the economic burden [14,15]. Thus, the development of improved AR techniques that enhance diagnostic accuracy while preserving the method’s inherent advantages (e.g., low-cost, non-invasiveness, and ease of use) would represent an advancement in the early detection and differentiation of middle ear fluid, particularly in paediatric and resource-limited clinical settings.

The eHEar project explores new methods to diagnose Otitis Media, such as using a smartphone as an AR device [16]. Our aim is to develop a device that integrates new technology with affordable hardware to provide improved diagnostic accuracy at an accessible price.

A new diagnostic instrument requires many rounds of testing and processing before it can be released to the market. Testing new medical instruments on patients can be very resource-intensive and time-consuming, which is why modelling is a powerful tool in product development and research. A model was built to replicate the test scenario and enable effective design iterations, allowing testing to be performed without the legal and practical challenges involved in testing prototypes on real patients. AR requires acoustic modelling, which can be divided into experimental measurement and virtual simulation. Experimental modelling has been well established for over a century, but simulation has only become feasible in recent decades as computational power has grown sufficiently. Studies have shown that acoustic simulations of the ear can reach very good agreement with reality [17,18].

Previous computational studies have modelled the middle ear using finite element methods to investigate sound transmission under conditions such as Otitis Media with Effusion [19,20]. These works demonstrated how varying fluid volumes affect tympanic membrane motion and hearing thresholds, providing insights into auditory mechanics. However, existing models primarily focus on transfer functions and hearing loss rather than diagnostic techniques, and none have simulated AR signals or assessed robustness to user variability. To the best of our knowledge, no prior study combines experimental AR measurements with finite element simulations to replicate the acoustic principles underlying middle ear fluid detection. We close this gap by creating and validating a combined experimental and computational approach for AR, making it possible to test different parameters and improve design for better accuracy and accessibility.

The purpose of this project was to develop both an experimental setup and a simulation model capable of replicating the acoustic principles used in detecting MEF via AR, with the aim of facilitating future research and technological advancements in AR. The simulation model was validated against measurements obtained from human individuals to ensure its reliability. A secondary objective was to evaluate the robustness of AR with respect to user variability through a systematic parameter study.

## 2. Theory

### 2.1. Acoustic Physics

**Figure 1:**
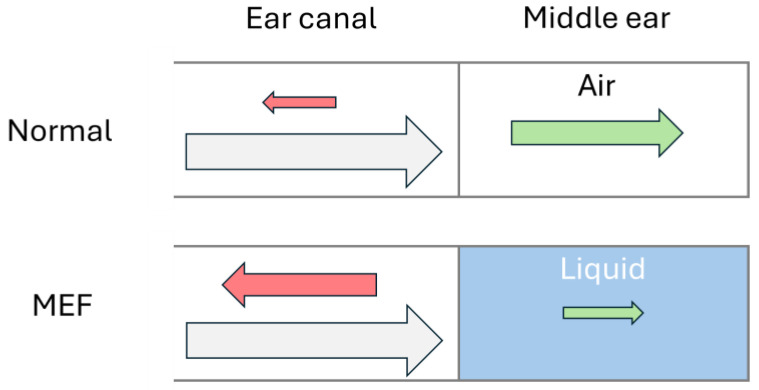
Schematic acoustics of a normal ear and an ear with middle ear fluid, where incoming sound waves (grey) are reflected (red) by the tympanic membrane or transferred (green) into the middle ear. The arrow size represents the amplitude of the sound wave.

TM mobility, a cornerstone in diagnosing OM, is detected indirectly through AR by emitting a chirp (playing a frequency sweep at constant amplitude) into the ear canal and measuring how much sound is reflected by the TM. A normal TM allows sound waves to pass into the middle ear and enter the inner ear via the ossicular bones in the middle ear. MEF restricts TM movement, causing it to reflect more sound back out through the ear canal, as illustrated in Figure 1. During recovery from OM, the eustachian tube often remains swollen and prevents ventilation of the middle ear. Poor ventilation can cause changes in the middle ear, leading to MEF with either retraction or bulging of the TM, both of which involve reduced compliance of the TM.

Resonance waves will appear when the sound wavelength (λ) and canal length (L) have the relationship described in Equation (1) [21], with the natural number n starting at 0 for the first resonance:

λ = 4L/(1 + 2n)(1)

**Figure 2:**
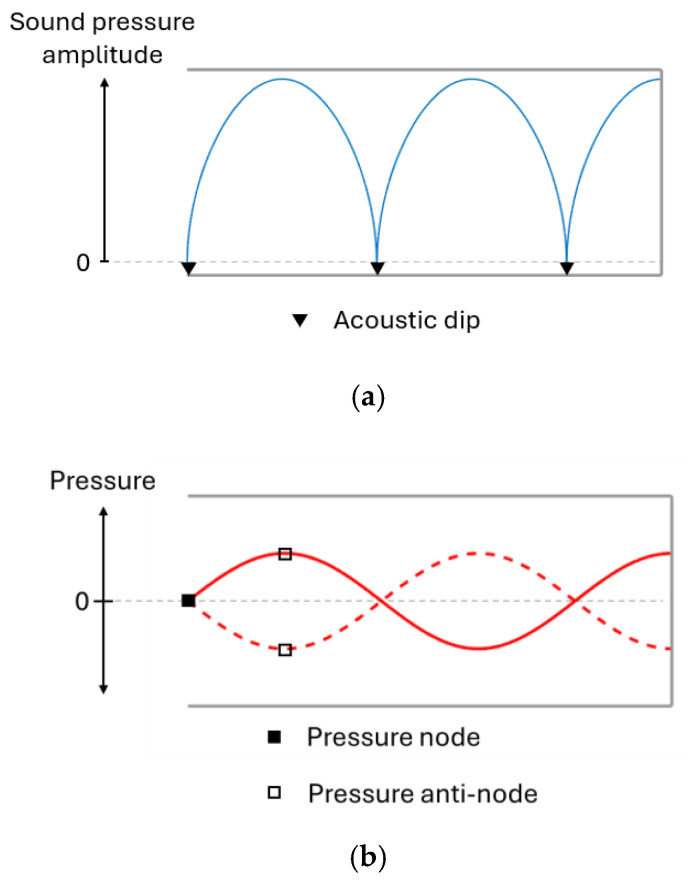
Sound pressure amplitude (**a**) and sound wave pressure (**b**) along the ear canal during resonance.

During resonance, the sound level will vary between different positions along the canal axis, as seen in Figure 2a. At certain positions, the sound pressure amplitude is doubled, while silent points (referred to as acoustic dips) are found in between.

**Figure 3:**
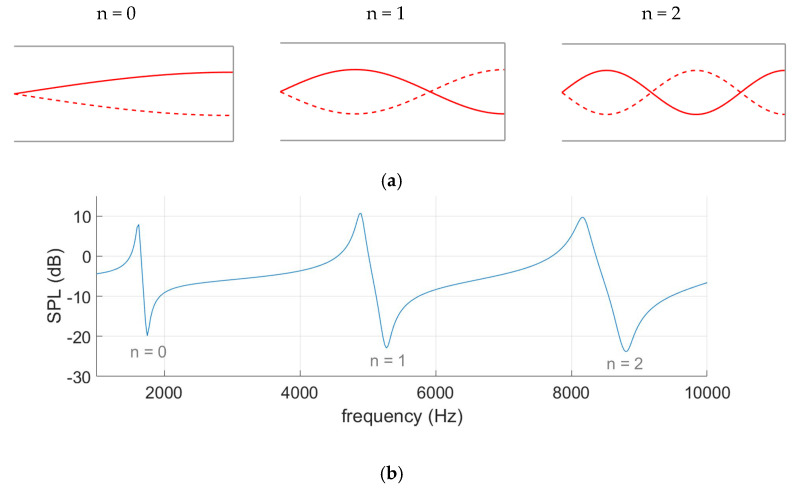
Pressure curves (**a**) illustrate the three resonances detected in the spectrum graph (**b**) of a tube with a closed end.

Resonance waves can be visualised through pressure curves, shown in Figure 3a, where an anti-node marks sound pressure oscillation with increased amplitude and the acoustic dips are found at nodes where pressure is static. All resonances produce an acoustic dip at the opening of the ear where AR measurements are made, as demonstrated in Figure 3b. When the frequency increases, so do the effects of the ear canal shape and the thermoviscous losses [22,23], which is why it is appropriate to analyse the first resonance.

Using Equation (1), the first resonance at a wavelength of 10 cm in an adult ear canal of 2.5 cm length can be predicted. Equation (2) translates that wavelength to 3430 Hz, using v = 343 m/s as the speed of sound through air:

f = v/λ (2)

### 2.2. Measurement Assessment

**Figure 4:**
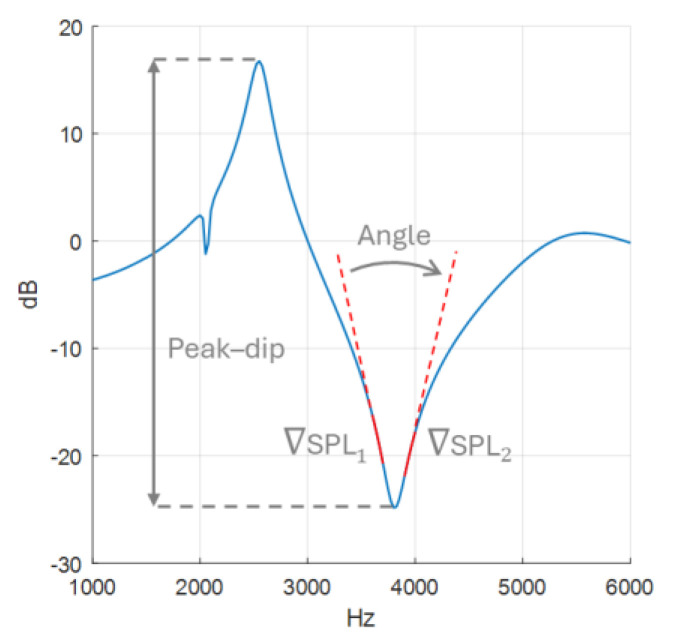
Spectral gradient angle with gradient SPL marked in solid red and peak–dip sound pressure level drawn out with a double-ended arrow.

AR measurements are presented in a spectrum graph, as seen in Figure 4, where sound pressure level (SPL) is plotted as a function of frequency. Spectral gradient angle (SGA) and peak–dip sound pressure level (SPL_peak-dip_) are utilised to evaluate the measurements. More reflection will increase the resonance, which will manifest itself as a lower SGA and a higher SPL_peak-dip_.

The SGA calculations, made in MATLAB 2023a, are based on a spectrum graph with 1 kHz per x-axis tick and 10 dB per y-axis tick. The angle is calculated from gradients ${\nabla SPL}_{1}$ and ${\nabla SPL}_{2}$, based on data points −200 to −100 Hz and 100 to 200 Hz relative to the frequency of the dip, as illustrated in Figure 4. These frequency intervals are chosen to provide SGA as they are as consistent as possible, as will be demonstrated later in the results of the sensitivity analysis. The gradients were calculated using the polyfit function, and to adjust for the chosen graph scale, 10 dB:1000 Hz, the y-values (dB) were multiplied by 100. Finally, SGA is extracted using Equation (3).

(3) $$SGA={cos}^{-1}\left(\frac{{\nabla SPL}_{1}\cdot {\nabla SPL}_{2}}{\left|{\nabla SPL}_{1}\right|\cdot |{\nabla SPL}_{2}|}\right)$$

## 3. Materials and Methods

In the following section, the components and building process of the experimental and simulation models will be presented. These will then be evaluated against each other and against patient samples. Finally, a sensitivity analysis will be performed using the simulation model to evaluate the robustness of SGA and SPL_peak-dip_.

### 3.1. Experiment Measurements

**Figure 5:**
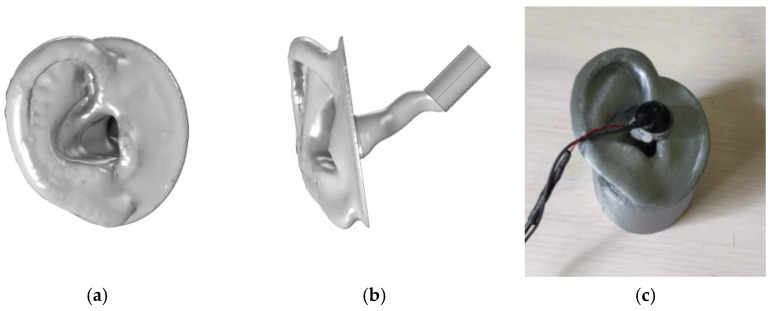
Presenting the ear model in digital form viewed from the side (**a**) and the front (**b**), and the 3D print (**c**) for the experiment.

The artificial ear type 4.3 by ITU [17,24] was selected as our ear representation, as shown in Figure 5. The geometry, which consisted of a pinna and ear canal, was built using a FDM 3D printer (Prusa i3 MK3S+, Prague, Czech Republic) with PLA. A cylinder 20 mm long and 10 mm in diameter was added to represent a normal adult middle ear volume of 1.1 ± 0.8 mL [25]. The model was completed with a supporting structure that encloses the canal and middle ear to facilitate the testing procedure. For the MEF case, the middle ear was filled with water, and a 0.08 mm thin latex sheet from a medical examination glove was mounted as an artificial TM.

The experimental measurements were carried out by placing the AR probe at the entry of the ear canal, where it emitted a chirp (frequency sweep) from 1 to 6 kHz. Five chirps were recorded for each measurement instance.

**Figure 6:**
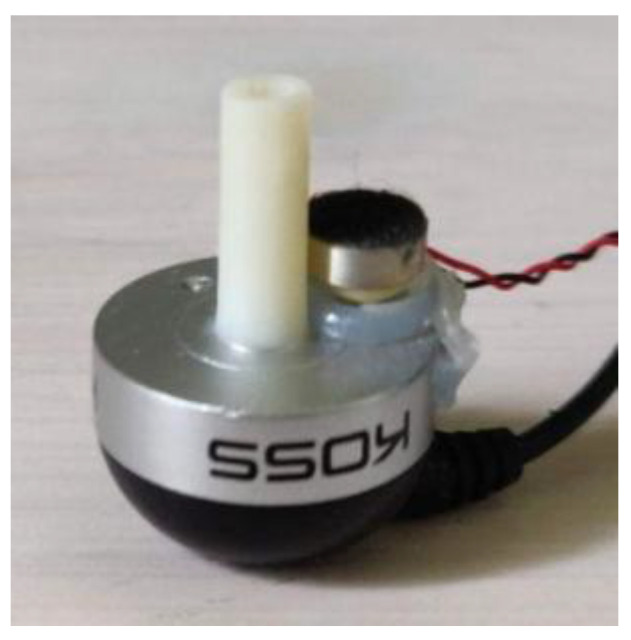
Probe with microphone attachment.

The probe was equipped with an in-ear headphone (Koss Sparkplugs, Koss Corporation, Milwaukee, USA) and a microphone (POM-2730L-HD-R, PUI Audio Inc., Fairborn, USA) attached to its base, as seen in Figure 6. The microphone was connected to a Roland QUAD-CAPTURE sound card (Roland Corporation, Hamamatsu, Japan).

The measurements were imported into MATLAB (R2023b, The MathWorks, Inc., Natick, MA, USA) and converted into sound pressure level (SPL_ear_), measured in decibels, as a function of frequency (f) through Fast Fourier Transform. For each instance, the five recordings were averaged into one measurement, which was then normalised using a reference SPL_ref_, as shown in Equation (4):

SPL(f) = SPL_ear_(f) – SPL_ref_(f) (4)

where the reference SPL_ref_ is a measurement taken with the probe directed into an open space. Finally, the data were presented in a spectrum graph, with SGA and SPL_peak-dip_ calculated as described previously.

### 3.2. Simulation Model

COMSOL Multiphysics 6.2 (COMSOL AB, Stockholm, Sweden) was used to perform the acoustic simulations on a laptop with a Ryzen 7 5800H 8-core CPU and 16 GB of RAM. The simulation time was approximately 30 min, with a 40k element mesh and frequency sweep from 0.5 to 8 kHz with a 25 Hz step resolution.

**Figure 7:**
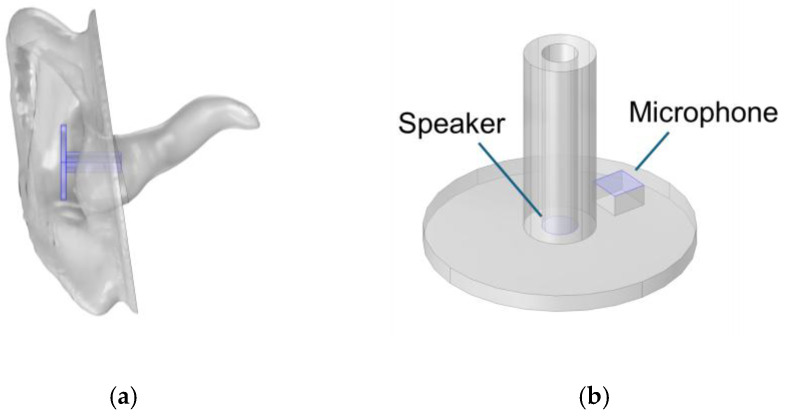
Geometry of the ear (**a**) and probe (**b**).

The same geometry of pinna and ear canal was used as in the experimental model, but the middle ear volume was simulated by applying a TM impedance representing the different states of the middle ear. A generic probe geometry was created using the COMSOL geometry editor, as shown in Figure 7.

To simulate acoustic physics, the Pressure Acoustics Frequency Domain was used, which utilises the Helmholtz equation, Equation (5), to solve for time-independent wave propagation. All symbols are defined in Table 1.

(5) $$\nabla \cdot \left(-\frac{1}{\rho }\left(\nabla p-q_{d}\right)\right)-\frac{\omega^{2}p}{c^{2}\rho }=Q_{m}$$

**Figure 8:**
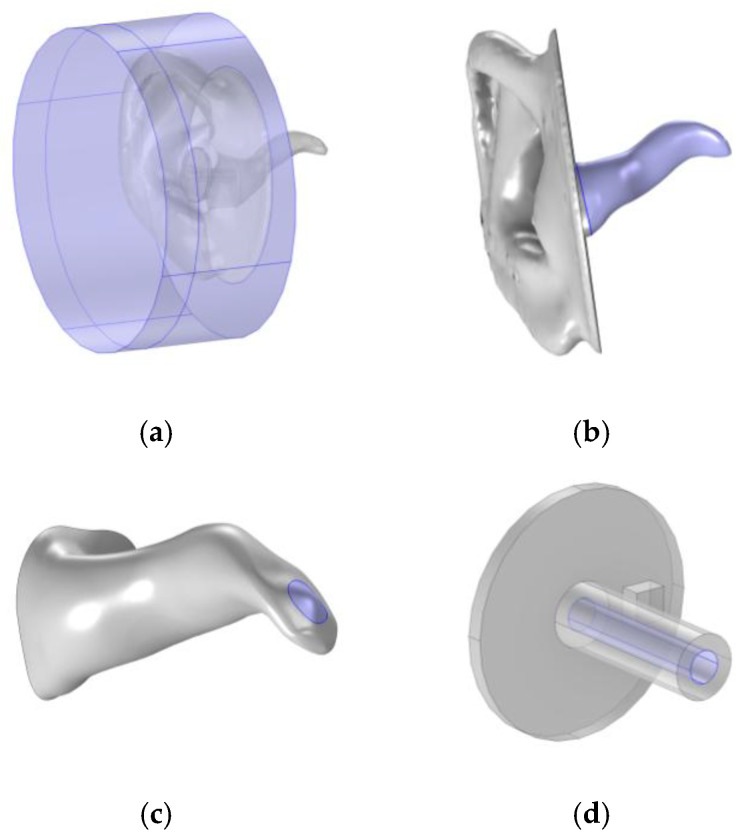
Described boundaries, highlighted in purple, on the cylinder domain (**a**), ear canal (**b**), tympanic membrane (**c**), and probe speaker channel (**d**).

Using the Type 4.3 ear simulator template from COMSOL, as in the works of L. B. Nielsen and M. Herring Jensen [17,18], the domain includes a cylinder with 90 mm diameter and 40 mm height around the ear pinna, presented in Figure 8a. Perfectly matched boundaries were applied to the cylinder surfaces to represent an atmospheric interface. The surfaces of the ear pinna and canal, presented in Figure 8b, and the probe were approximated as hard walls, i.e., reflective surfaces, while the TM, marked in Figure 8c, was assigned an impedance that varied with frequency [26]. The speaker membrane was set as a normal velocity boundary, defined as a harmonically oscillating surface with a normal velocity of 0.01 m/s over an area of 3.13 mm^2^. Additionally, a specific “thermoviscous losses” boundary condition was applied on the surfaces of the speaker channel and ear canal, presented in Figure 8b,d, to account for the friction that occurs as the soundwaves force air to move along the walls [22].

**Figure 9:**
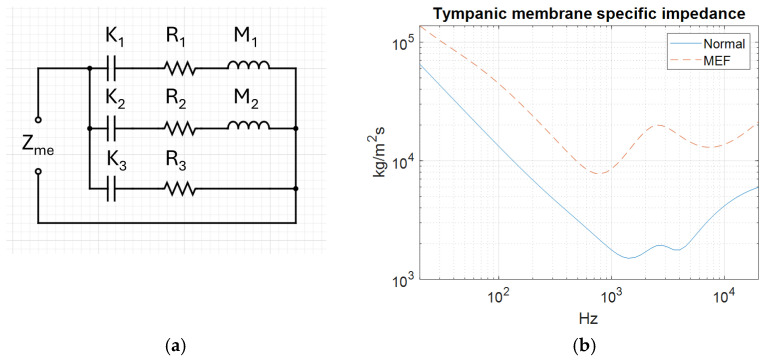
Electric analogue model (**a**) and tympanic membrane impedance (Z_me_) by frequency for normal and middle ear fluid conditions (**b**).

To reproduce the acoustic behaviour of MEF and a healthy middle ear it is important to assign the correct physical properties at the TM where the ear canal and middle ear interface. The acoustic impedance decides the mobility of the TM. Higher impedance results in more reflection; a stronger resonance of the ear canal yields a more pronounced acoustic dip. The impedance values presented in Figure 9b were not calculated in this study but were taken from a previously published electrical analogue model [26]. In that model, the impedance was obtained by adjusting the values of the electrical components (representing mass, stiffness, and resistance) to match measured acoustic responses from 18 patients with MEF and 9 healthy individuals.

The mesh element size determines the level of detail in the simulation model. Our simulation uses a physics-based meshing algorithm, which determines element size based on the wavelength of interest. The shortest wavelength in the simulation was 42.9 mm at 8 kHz, given by Equation (2). To resolve a wave in an acoustic simulation, element size must be no larger than one-fourth of its wavelength, i.e., 10.7 mm. 

**Figure 10:**
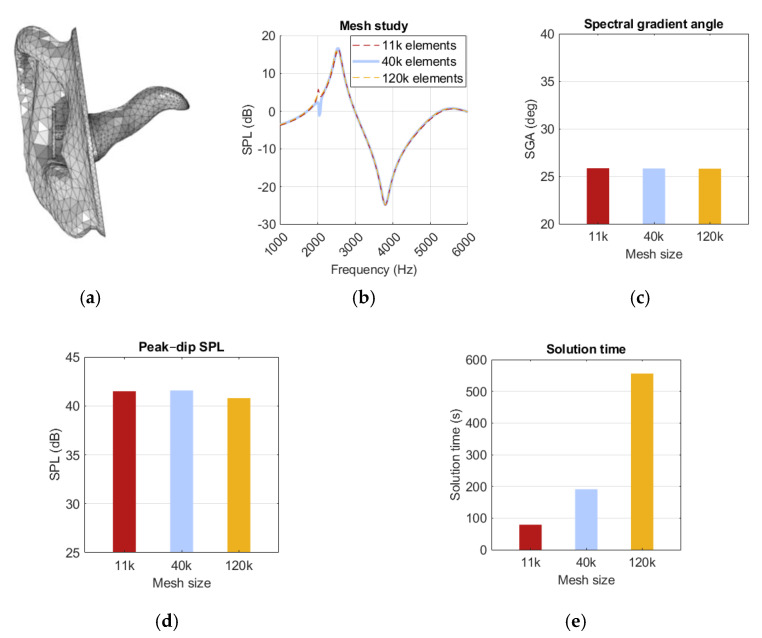
Illustration of (**a**) a mesh model with 40k elements, (**b**) a spectrum graph, (**c**) spectral gradient angle (SGA), (**d**) peak–dip sound pressure level (SPL), (**e**) solution time for the 11k-, 40k-, and 120k-element meshes.

The mesh generated under these conditions consisted of 40k elements, as shown in Figure 10d. To ensure sufficient mesh resolution, comparisons were made with a coarser and a finer mesh, with element counts ranging from 11k to 120k. To select a coarser mesh, results would need to be preserved while improved simulation time was achieved. The 11k mesh did perform more than twice as fast as the 40k mesh, but because of frequent issues in resolving fine geometry details and meshing errors, it was excluded as an option. The refined mesh needed to show a significant difference from the original mesh to be relevant, but it showed only minor changes in both the peak–dip amplitude and the SGA, as presented in Figure 10b,c. A notch at 2 kHz was observed in all meshes, but it did not exhibit any trend with mesh refinement, as seen in Figure 10a. Thus, the default mesh was kept unchanged, as no significant advantage was offered by any of the alternative meshes. 

Sound pressure level data were collected by the microphone every 25 Hz from 0.5 to 8 kHz. The data were imported into MATLAB and normalised using measurements from a simulation run without an ear into an open volume. SGA and SPL_peak-dip_ were calculated as in the experimental model. The accuracy of the simulation model was assessed by comparing the results with measurements from both the experimental model and the patient samples.

### 3.3. Patient Samples

For verification, sample data from a healthy volunteer and a patient with AOM were used. The sample measurements were collected with the same recording equipment and protocol as for the experimental measurements. The healthy ear sample was collected from a 37-year-old male with no significant history of ear infections or abnormalities. The AOM sample was collected at Backens hälsocentral in Umeå on a 17-year-old male with bilateral AOM who had started antibiotic treatment 3 days prior to the visit, but without improvement. The symptoms presented were pounding ear pain and impaired hearing. The diagnosis was confirmed by a primary care resident physician using pneumatic otoscopy, which revealed a bulging and bright pink opaque TM with low mobility during pneumatic provocation.

### 3.4. Sensitivity Analysis of Probe Orientation

**Figure 11:**
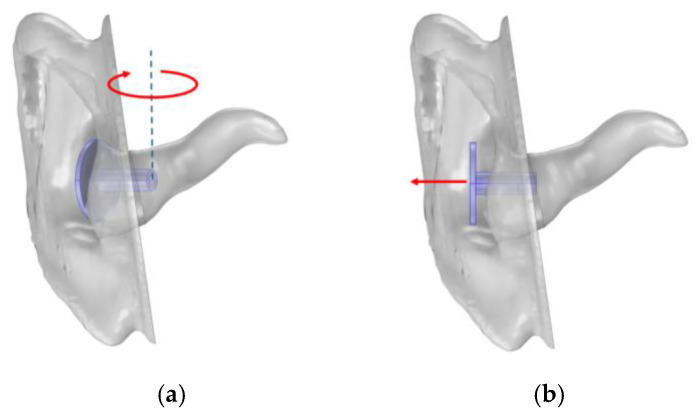
Illustration of (**a**) probe rotation towards the front of the ear canal and (**b**) offset from the ear.

It is challenging to ensure measurement consistency between users with handheld instruments. If the equipment is sensitive to variations in position and angle, there is an imminent risk that the result will be misleading. To assess robustness against user error, the simulation model was used to investigate how the SGA and SPL_peak-dip_ were affected by varied rotation and position of the probe. Rotation was investigated with different angles around the vertical axis, and position was varied with offset along the axis of the ear canal, as visualised in Figure 11. Polynomials are presented to describe the SGA and SPL_peak-dip_ as functions of probe rotation or position if they had a significant impact on the result.

## 4. Results

The sampling, experiments, and simulations were compared by examining SGA and SPL_peak-dip_ for a normal ear and for MEF conditions. Lastly, the impact of variation in probe rotation and offset on the results was investigated using the simulation model.

### 4.1. Sample and Simulation with Normal Ear 

**Figure 12:**
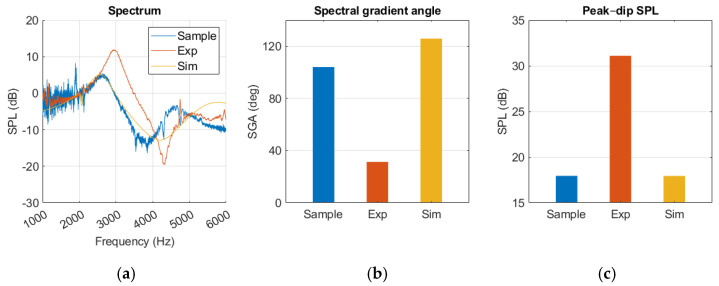
Normal ear represented in sample, experiment (Exp), and simulation (Sim) in a (**a**) spectrum graph, (**b**) spectral gradient angle (SGA) diagram, (**c**) peak–dip sound pressure level (SPL) diagram.

**Figure A1:**
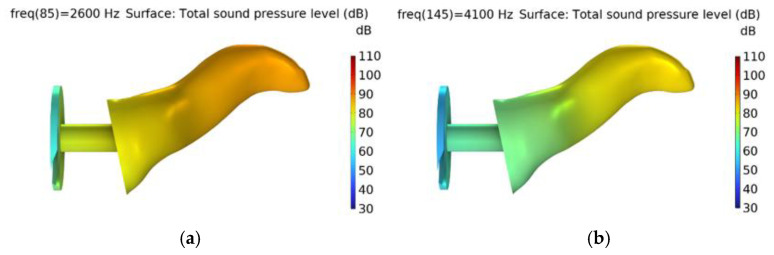
Normal ear with total sound pressure level distribution at (**a**) peak (2.6 kHz) and (**b**) dip (4.1 kHz).

The results presented in Figure 12 revealed that the experimental model produced a sharper SGA than the sample and simulation. Additionally, the SPL_peak-dip_ was higher, indicating greater reflection at the TM. The sample and simulation results were closer in agreement, although the SGA was 22 degrees lower for the sample. Above 4.5 kHz there was a clear discrepancy between the curves, where the sample curve started falling before the simulation reached its second peak. The sample spectrum curve showed a significant amount of noise despite averaging five measurements. In Figure A1, the simulation results present similar pressure distribution at peak and dip frequencies, where pressure was lowest at the canal opening and gradually increased towards the TM.

**Figure 13:**
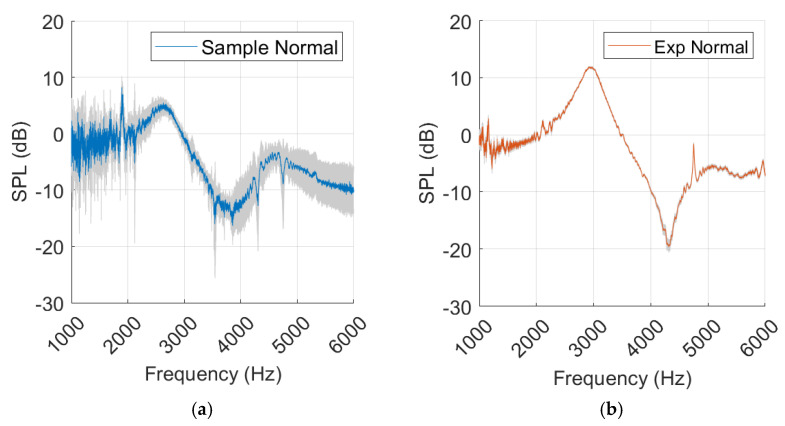
Sample measurements from normal ear (**a**) and experimental (Exp) values (**b**), with standard deviation marked in grey.

The sample measurements present more standard deviation than the experimental measurements, as shown in Figure 13.

### 4.2. Sample, Experiment, and Simulation with MEF

**Figure 14:**
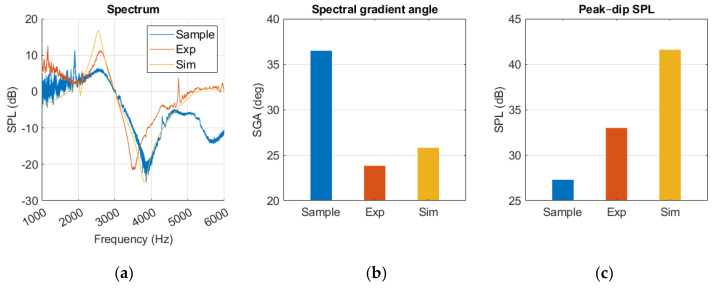
Middle ear fluid measurements from the sample, experiment (Exp), and simulation (Sim) in a (**a**) spectrum graph, (**b**) spectral gradient angle (SGA) diagram, (**c**) peak–dip sound pressure level (SPL) diagram.

In Figure 14, a resonance 400 Hz lower than that of the sample measurement was produced by the experimental model. The gradient was sharper around the dip, and the SPL_peak-dip_ was larger for the experimental model. After 5 kHz there was a deviation between the curves, where a secondary dip occurred in the sample measurement. Both measurements were affected by noise at frequencies below 2 kHz.

Like our experimental measurements, the simulation presented a sharper SGA, a higher SPL_peak-dip_, and lacked the secondary dip when compared to the sample. Resonance occurred within 100 Hz of the sample results. A notch at 2 kHz was observed; however, it appeared as negative rather than positive, in contrast to the sample and experimental measurements. 

**Figure A2:**
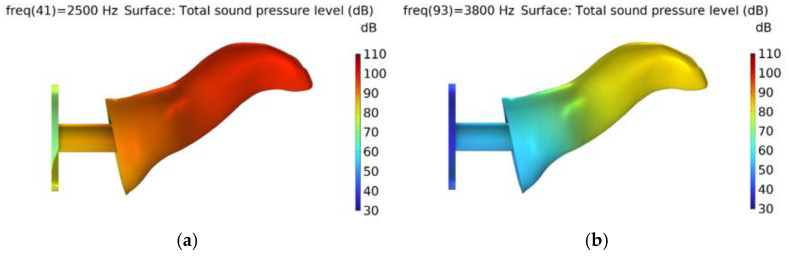
Middle ear fluid scenario with total sound pressure level distribution at (**a**) peak (2.5 kHz) and (**b**) dip (3.8 kHz).

Compared to the healthy ear, there was an overall pressure increase along the ear canal, as seen in Figure A2. However, the distribution was similar, with low pressure at the canal opening and higher pressure at the TM.

There was relative similarity between the simulation and the experimental model after 2 kHz. The resonance frequency occurred at a slightly higher frequency and had a higher SPL_peak-dip_, but the curve development followed a similar path.

There was a significant difference in SGA and peak–dip between the normal and MEF results in both the simulation and samples.

**Figure 15:**
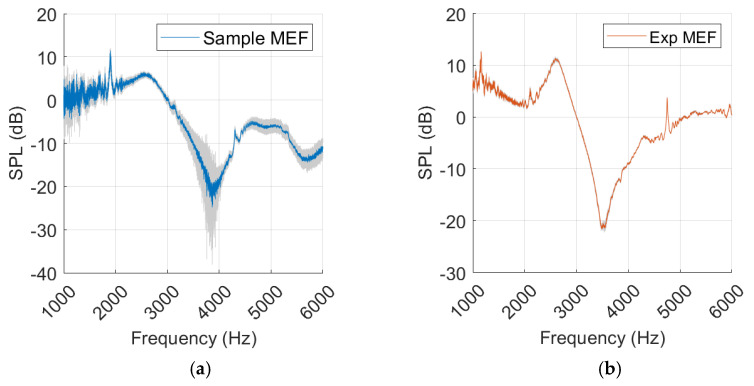
Measurements with middle ear fluid from sample (**a**) and experiment (Exp) (**b**), with standard deviation marked in grey.

Standard deviation is most prominent around the resonance frequency in the sample, while it is relatively subtle and consistent in the experimental measurements, as shown in Figure 15.

### 4.3. Simulation with Probe Rotation and Offset

**Figure 16:**
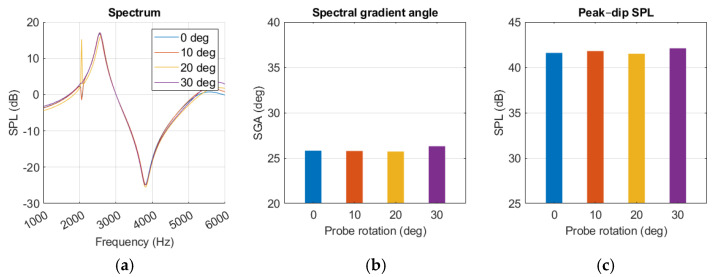
Middle ear fluid simulation with variations in probe angle presented as (**a**) spectrum graph, (**b**) spectral gradient angle, (**c**) peak–dip sound pressure level (SPL) chart.

The probe angle variations had little effect on the resonance behaviour, as shown in Figure 16a. The notch at 2 kHz was affected but did not show any trends with increased probe rotation. SGA remained within a few degrees as the probe rotation changed. Amplitude increased with more rotation at frequencies above 5 kHz, but around the peak and dip it remained within a few decibels. No polynomials were calculated for either SGA or SPL_peak-dip_ level because of the low impact of probe rotation.

**Figure 17:**
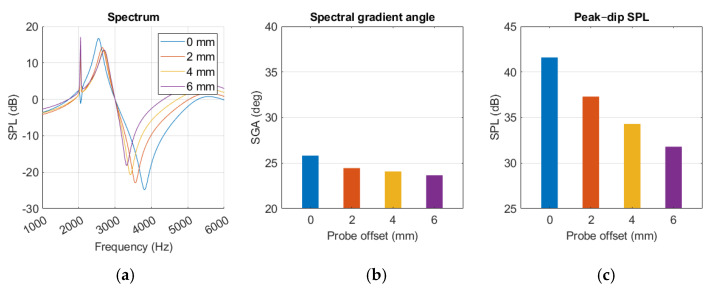
MEF simulation with variations in probe offset position, presented as (**a**) spectrum graph, (**b**) spectral gradient angle, (**c**) peak–dip sound pressure level chart.

The dip occurs at a lower frequency with increased offset, while the peak remains more stationary, as seen in Figure 17a. The notch at 2 kHz was quite pronounced for some of the offsets and intensified with increasing offset distance.

Probe offset appeared to have a small decreasing effect on SGA and a more prominent impact on SPL_peak-dip_, described with Equation (6) and (7), respectively.

SPL_peak-dip_ = −0.0167x^3^ + 0.2625x^2^ – 2.6083x + 41.600 (6)

SGA = −0.0218x^3^ + 0.2556x^2^ – 1.1087x + 25.8209 (7)

where x is the offset distance. The diagrams in Figure 17b,c show decreasing trends in SGA and SPL_peak-dip_ with growing offset. Moving the probe from a 0 to 6 mm offset resulted in SGA decreasing by 2.2 degrees and SPL_peak-dip_ reducing by 9.8 dB.

## 5. Discussion

In this study, an acoustic computational simulation and a 3D-print-based experiment were developed to replicate human AR measurements, showing promising resemblance. The sensitivity study demonstrated the efficiency and versatility of working with simulations, establishing how SGA and SPL_peak-dip_ were affected by probe position but not rotation. Conducting a parameter study (i.e., changing ear canal geometry or TM impedance) took only a few minutes in the simulation model, whereas doing so in an experimental approach could require hours of work. We believe this simulation model has potential to accelerate progress in AR diagnostics.

To our knowledge, there is no standard protocol to calculate the SGA, which prevents a comparison of SGA values with those reported by Lindén [10], as it cannot be confirmed that the same graph scales were used. However, our results showed resemblance to the typical MEF spectral graph (type 4) of the EarCheck Pro [10]. For this study, a simple side-by-side comparison with spectrum graphs of previous studies provided sufficient insight to confirm that the resonance was captured correctly. The use of different AR devices and study sample patients renders comparison of SGA and graph values from other studies purposeless. Once the diagnostic accuracy of the device is investigated, it will be more meaningful to compare our results with other studies.

The healthy ear showed good agreement between sample and simulation peak–dip values, while SGA was less similar, as shown in Figure 12. The experimental results for the healthy ear were found to be unsatisfactory, and the same level of agreement between the experiment and the simulation as reported by Nielsen was not achieved [17]. Nevertheless, this was somewhat expected considering the rough approximation used for our TM material. Due to limited resources, this source of error in the TM was accepted. In the MEF case, a closer similarity was observed, along with a clear resonance amplification in the spectrum curves of the simulation, experiment, and sample. The added impedance of the middle ear fluid overshadowed the effect of an inaccurate TM in the experiment, which may explain the improved agreement.

Higher SPL_peak-dip_ and lower SGA relative to the sample in the MEF case indicated that there were neglected energy losses in our simulation and experiment. The effect of the impedance at the TM and canal surface will be relevant to investigate further in order to achieve sample agreement with the experiment and simulation. The 3D-printed PLA, which is a hard plastic, and the simulation boundary were both defined as a hard surface with complete sound reflection. It is considered reasonable that a softer material, such as human skin, would absorb more energy than the surface approximations used in both the experiment and simulation. Different surface material configurations should be investigated to establish the impact of these factors.

The experimental model showed limited agreement with the normal ear measurements, particularly for SGA. Several factors likely contributed to this mismatch, including the use of 3D-printed PLA for the ear canal and a simplified TM material. Both the PLA canal and TM approximation were treated as rigid surfaces, neglecting acoustic damping and energy absorption present in human tissue. Increasing the absorption of the TM is expected to reduce reflected energy in the ear canal, which would likely decrease SPL_peak-dip_ values and increase SGA, as more acoustic energy is dissipated rather than reflected. For instance, if the TM material were modelled with a damping coefficient closer to physiological tissue, the sharpness of spectral peaks in the SPL would be reduced, resulting in smaller peak–dip differences, while SGA (which integrates spectral amplitude variations) would increase slightly due to the smoother frequency response. Such adjustments would likely improve agreement between simulation and experimental measurements of the normal ear, particularly in frequency regions where the rigid TM assumption currently overestimates reflection. Potential improvements include using softer, more compliant TM materials, incorporating acoustic damping layers in the canal, or refining the geometry to better match the human ear. Although the quantitative impact of these approximations on outcome measures has not been assessed, the observed discrepancies indicate that absolute values for SGA and SPL_peak-dip_ should be interpreted cautiously. Despite these limitations, the overall trends and sensitivity of the simulation to probe placement were maintained, demonstrating that the model remains valuable for relative comparisons and methodological development.

AOM is not a binary condition, i.e., an infected middle ear will have a gradual transition into a normal state, often including OME and an under-pressure middle ear as an intermediate state. As it heals, the impedance will decrease at the TM and measurements will develop into a healthy curve profile. Because the severity of the sample AOM could not be objectively determined, it remains unclear whether reflection was overpredicted by the experiment and simulation, or if the sample was partially healed. The sample measurement displayed a second dip at 5 to 6 kHz, which neither simulation nor experiment was able to reproduce. The presence of a middle ear air pocket, allowing sound waves to travel through the middle ear and produce an auxiliary resonance, may explain the second dip in the spectrum. A simulation or experimental model with a partially filled middle ear would be required to determine whether this hypothesis is plausible. This is supported by studies showing that variation in middle ear volume has a significant effect on ear canal reflection [27]. Additionally, the ear canal depth and shape are factors likely to influence resonance behaviour, and because the ear canal anatomy of children differs from adults, it would be appropriate to proceed with analysis of a child’s ear geometry and samples from paediatric patients. CSOM was not investigated, but should be included in future research, as a perforated TM is expected to produce a different footprint in AR measurements compared to MEF or negative middle ear pressure. According to Lindén, the EarCheck Pro provides spectrum graphs representing negative middle ear pressure without MEF, suggesting that AR has potential to distinguish between TM immobility caused by MEF or negative pressure. A sensitivity analysis with varied middle ear pressure could bring useful insight in how to differentiate between causes of TM immobility with AR.

Similarly to the findings of L. Nielsen [17], variations in simulation results were observed from small changes in probe placement. Maintaining probe position was more challenging during human measurement, possibly explaining the increase in standard deviation compared to the experiment, as shown in Figure 13 and Figure 15. The sensitivity analysis suggested that SGA, described with Equation (6), remained relatively stable with increasing probe rotation, while SPL_peak-dip_ and resonance frequency displayed falling trends, as in Figure 17 and Equation (7). Probe rotation presented little effect on any of our key values. Because sound waves are uniformly distributed spherically from a point source, similar results are expected when the probe is rotated in any direction within 30 degrees.

Although all measurements were affected by noise below 2 kHz, a clear distinction in SGA and SPL_peak-dip_ was observed when comparing MEF to the normal ear in human individuals, supporting the potential of low-cost AR equipment to detect MEF. The presence of noise makes it difficult to determine whether the notch at 2 kHz is an artefact or an acoustic phenomenon, and measurements with higher-quality equipment could help clarify this.

A key limitation of this study is that validation was performed on only one healthy ear and one ear with AOM. While these cases demonstrate the feasibility of the proposed approach, they are not sufficient to draw generalizable conclusions regarding performance across broader patient populations. Therefore, the present work should be regarded as a proof-of-concept demonstration, motivating future studies involving a larger and more diverse cohort to robustly assess reliability and clinical applicability.

## 6. Conclusions

The results showed that both experimental and simulation models successfully reproduce the resonance phenomenon caused by MEF, offering valuable support in developing acoustic reflectometric instruments and insights into anatomical and pathological variations. Overpredictions in both models suggest that assumptions regarding energy loss and absorption by the skin and TM require refinement to improve agreement with patient measurements. The sensitivity analysis indicated reduced resonance frequency and SPL_peak-dip_ with increasing probe offset, while probe rotation had minimal effect within 30 degrees, supporting the robustness of SGA. The effective use of simple, low-cost probe components supports the feasibility of building affordable devices, which could be particularly valuable in addressing OM-related hearing loss in developing regions. Future work should investigate paediatric scenarios, boundary conditions for partially filled middle ears, perforation, and negative middle ear pressure to clarify measurement transitions from MEF to normal states.

## Figures and Tables

**Table 1:** Description of symbols in Helmholtz wave equation.

Symbol	Description	Unit
ρ	Density of continuum (air)	kg/m^3^
p	Pressure	Pa
$q_{d}$	Dipole domain source (speaker)	N/m^3^
$Q_{m}$	Monopole domain source (background)	1/s
ω	Angular frequency	rad/s
c	Speed of sound in continuum (air)	m/s

## Data Availability

Data collected from human individuals are unavailable due to privacy and ethical restrictions.

## Abbreviations

The following abbreviations are used in this manuscript:

AOM	Acute Otitis Media
AR	Acoustic Reflectometry
CSOM	Chronic Suppurative Otitis Media
MEF	Middle Ear Fluid
OM	Otitis Media
OME	Otitis Media with Effusion
TM	Tympanic Membrane

## References

[1] Monasta L., Ronfani L., Marchetti F., Montico M., Brumatti L.V., Bavcar A., Grasso D., Barbiero C., Tamburlini G. (2012). Burden of Disease Caused by Otitis Media: Systematic Review and Global Estimates. PLoS ONE.

[2] Bhatia R., Chauhan A., Rana M., Kaur K., Pradhan P., Singh M. (2024). Economic Burden of Otitis Media Globally and an Overview of the Current Scenario to Alleviate the Disease Burden: A Systematic Review. Int. Arch. Otorhinolaryngol..

[3] Blomgren K., Pitkäranta A. (2003). Is it possible to diagnose acute otitis media accurately in primary health care?. Fam. Pract..

[4] Gisselsson-Solen M. (2015). Acute Otitis Media in Children—Current Treatment and Prevention. Curr. Infect. Dis. Rep..

[5] Sundvall P.-D., Papachristodoulou C.E., Nordeman L. (2019). Diagnostic methods for acute otitis media in 1 to 12 year old children: A cross sectional study in primary health care. BMC Fam. Pract..

[6] Azevedo C., Machado J.F., Lima A.F., Mar F.M., Vilarinho S., Dias L. (2022). Value of simple otoscopy in diagnosing otitis media with effusion in children. Acta Otorrinolaringol. Engl. Ed..

[7] Watters G., Jones J., Freeland A. (1997). The predictive value of tympanometry in the diagnosis of middle ear effusion. Clin. Otolaryngol..

[8] Merchant G.R., Al-Salim S., Tempero R.M., Fitzpatrick D., Neely S.T. (2021). Improving the Differential Diagnosis of Otitis Media With Effusion Using Wideband Acoustic Immittance. Ear Hear..

[9] Lee H.Y., Choi M.S., Park N.S., Cho C.S. (2014). Applicability of spectral gradient acoustic reflectometry (EarCheck): Screening patients who need surgical treatment for chronic otitis media with effusion. Int. J. Pediatr. Otorhinolaryngol..

[10] Lindén H., Teppo H., Revonta M. (2006). Spectral gradient acoustic reflectometry in the diagnosis of middle-ear fluid in children. Eur. Arch. Oto-Rhino-Laryngol..

[11] Muderris T., Yazıcı A., Bercin S., Yalçıner G., Sevil E., Kırıs M. (2013). Consumer acoustic reflectometry: Accuracy in diagnosis of otitis media with effusion in children. Int. J. Pediatr. Otorhinolaryngol..

[12] Babb M.J., Hilsinger R.L., Korol H.W., Wilcox R.D. (2004). Modern Acoustic Reflectometry: Accuracy in Diagnosing Otitis Media with Effusion. Ear, Nose Throat J..

[13] Chianese J., Hoberman A., Paradise J.L., Colborn D.K., Kearney D., Rockette H.E., Kurs-Lasky M. (2007). Spectral Gradient Acoustic Reflectometry Compared with Tympanometry in Diagnosing Middle Ear Effusion in Children Aged 6 to 24 Months. Arch. Pediatr. Adolesc. Med..

[14] Erkkola-Anttinen N., Laine M.K., Tähtinen P.A., Ruohola A. (2015). Parental Role in the Diagnostics of Otitis Media: Can Layman Parents Use Spectral Gradient Acoustic Reflectometry Reliably?. Int J Pediatr Otorhinolaryngol.

[15] Teppo H., Revonta M. (2009). Consumer acoustic reflectometry by parents in detecting middle-ear fluid among children undergoing tympanostomy. Scand. J. Prim. Heal. Care.

[16] Chan J., Raju S., Nandakumar R., Bly R., Gollakota S. (2019). Detecting middle ear fluid using smartphones. Sci. Transl. Med..

[17] Nielsen L.B. (2023). Simulation and Physical Testing Using Standardized Ear Simulators. DAGA Hambg..

[18] Nielsen L.B. (2022). The Digital Twin of a New and Standardized Fullband Ear Simulator. DAGA Hambg..

[19] Gan R.Z., Wang X. (2007). Multifield coupled finite element analysis for sound transmission in otitis media with effusion. J. Acoust. Soc. Am..

[20] Areias B., Parente M.P.L., Santos C., Gentil F., Jorge R.M.N. (2017). The human otitis media with effusion: A numerical-based study. Comput. Methods Biomech. Biomed. Eng..

[21] Wu C., Chen L., Ni J., Xu J. (2016). Modeling and experimental verification of a new muffler based on the theory of quarter-wavelength tube and the Helmholtz muffler. SpringerPlus.

[22] Berggren M., Bernland A., Noreland D. (2018). Acoustic boundary layers as boundary conditions. J. Comput. Phys..

[23] Lewis J.D. (2018). The area discontinuity between probe and ear canal as a source of power-reflectance measurement-location variability. J. Acoust. Soc. Am..

[24] COMSOL AB (2021). Artificial Ears, 2021.

[25] Ahn J.-Y., Park H.J., Park G.-H., Jeong Y.-S., Kwak H.-B., Lee Y.-J., Shin J.-E., Moon W.-J. (2008). Tympanometry and CT Measurement of Middle Ear Volumes in Patients with Unilateral Chronic Otitis Media. Clin. Exp. Otorhinolaryngol..

[26] Merchant G.R., Neely S.T. (2021). The influence of otitis media with effusion on middle-ear impedance estimated from wideband acoustic immittance measurements. J. Acoust. Soc. Am..

[27] Voss S.E., Horton N.J., Woodbury R.R., Sheffield K.N. (2008). Sources of Variability in Reflectance Measurements on Normal Cadaver Ears. Ear Hear..

